# Plant-Produced Anti-Zika Virus Monoclonal Antibody Glycovariant Exhibits Abrogated Antibody-Dependent Enhancement of Infection

**DOI:** 10.3390/vaccines11040755

**Published:** 2023-03-29

**Authors:** Ming Yang, Haiyan Sun, Huafang Lai, Biswas Neupane, Fengwei Bai, Herta Steinkellner, Qiang Chen

**Affiliations:** 1 The Biodesign Institute and School of Life Sciences, Arizona State University, Tempe, AZ 85225, USA; 2 Department of Cell and Molecular Biology, University of Southern Mississippi, Hattiesburg, MS 39406, USA; 3 Department of Applied Genetics and Cell Biology, University of Natural Resources and Life Sciences, 1180 Vienna, Austria

**Keywords:** Zika virus, monoclonal antibody (mAb), plant-made antibody, antibody dependent enhancement of infection (ADE), antibody-dependent cellular cytotoxicity (ADCC), Fc effector function, glycosylation, neutralization, plant-made pharmaceutical

## Abstract

Monoclonal antibodies (mAb) against the envelope (E) protein of Zika virus (ZIKV) have shown great potential as therapeutics against the Zika epidemics. However, their use as a therapy may predispose treated individuals to severe infection by the related dengue virus (DENV) via antibody-dependent enhancement of infection (ADE). Here, we generated a broadly neutralizing flavivirus mAb, ZV1, with an identical protein backbone but different Fc glycosylation profiles. The three glycovariants, produced in wild-type (WT) and glycoengineered ΔXF *Nicotiana benthamiana* plants and in Chinese hamster ovary cells (ZV1^WT^, ZV1^ΔXF^, and ZV1^CHO^), respectively, showed equivalent neutralization potency against both ZIKV and DENV. By contrast, the three mAb glycoforms demonstrated drastically different ADE activity for DENV and ZIKV infection. While ZV1^CHO^ and ZV1^ΔXF^ showed ADE activity upon DENV and ZIKV infection, ZV1^WT^ totally forwent its ADE. Importantly, all three glycovariants exhibited antibody-dependent cellular cytotoxicity (ADCC) against virus-infected cells, with increased potency by the fucose-free ZV1^ΔXF^ glycoform. Moreover, the in vivo efficacy of the ADE-free ZV1^WT^ was demonstrated in a murine model. Collectively, we demonstrated the feasibility of modulating ADE by Fc glycosylation, thereby establishing a novel approach for improving the safety of flavivirus therapeutics. Our study also underscores the versatile use of plants for the rapid expression of complex human proteins to reveal novel insight into antibody function and viral pathogenesis.

## 1. Introduction

Zika virus (ZIKV) is a mosquito-vectored flavivirus and is closely related to other members of the *Flaviviridae* family, including the four serotypes of dengue virus (DENV), West Nile virus (WNV), Japanese encephalitis virus (JEV), yellow fever virus (YFV), and tick-borne encephalitis virus (TBEV) [1]. Most ZIKV infections in humans lead to self-limiting febrile illnesses of short duration with symptoms including rash, headache, and myalgia. In recent years, however, ZIKV has attracted worldwide attention due to its link with the development of severe fetal abnormalities, including microcephaly, as well as neurological disorders in adults, such as Guillain-Barré syndrome [2,3,4]. Due to these severe manifestations and the lack of licensed vaccines [5], there is an urgent need to develop effective and safe therapeutics to treat ZIKV infection.

The envelope (E) protein of ZIKV mediates host cellular recognition, attachment, and the subsequent membrane fusion for viral entry [1,6,7]. The E protein shares the typical three-domain structure (EDI, EDII, and EDIII) with other flaviviruses and is a major target of host humoral responses [1,8]. For example, potent antibody responses against EDII and EDIII have been reported in naturally infected patients or in subjects administered with E protein-based vaccines [9,10,11,12,13]. As neutralizing antibody responses are the major correlate of protection for licensed vaccines against YFV and TBEV and are found to be protective against infection by many other flaviviruses [11,14,15], monoclonal antibodies (mAb) against the E protein have been considered as strong candidates for ZIKV therapeutics.

However, the development of mAb-based therapies for ZIKV faces several challenges, including the potential risk of inducing antibody-dependent enhancement (ADE) of DENV infection due to the genetic similarity between the two viruses [16]. ADE has been demonstrated clinically during a secondary DENV infection by a new DENV serotype due to the presence of non-neutralizing or sub-neutralizing antibodies from the primary infection [17]. Instead of neutralizing the new serotype of DENV, these antibodies form complexes with DENV that bind to Fc gamma receptor (FcγR)-bearing myeloid cells, resulting in increased viral uptake and replication, leading to a potentially lethal dengue hemorrhagic fever/dengue shock syndrome (DHF/DSS) [18,19]. As ZIKV and DENV are closely related and co-circulate geographically, ZIKV mAb therapeutics against the common epitopes of ZIKV and DENV may have the potential to trigger ADE in treated patients when they are secondarily exposed to DENV. In fact, enhancement of DENV infection and disease symptoms by treatment with anti-ZIKV antibodies have been reported both in cell culture and in mice [20,21,22,23]. Therefore, eliminating the risk of ADE for DENV infection should be a critical consideration for the development of ZIKV therapeutics.

The binding of the antibody-DENV complex to FcγRs on the surface of myeloid cells is essential for FcγR-mediated ADE to occur [17]. In turn, the binding of IgG antibodies to FcγRs depends on the presence and the composition of glycans on the single conserved N-glycosylation site at the IgG Fc domain [24,25]. Therefore, it is possible to modulate the ADE activity of an anti-ZIKV mAb by regulating the mAb-FcγRs interaction via controlling mAb N-glycosylation. Here, we used ZV1, a mAb recognizing a common epitope conserved on the EDII fusion loop of ZIKV, DENV, WNV, and YFV [8,26], as an example to investigate the impact of Fc-glycosylation on ADE activity. ZV1 was produced in Chinese hamster ovary (CHO) cells and in wild-type (WT) and ∆XF *Nicotiana benthamiana*, a glycoengineered RNAi plant line with downregulated genes for the synthesis of xylose and core fucose [24]. Plant-produced ZV1 exhibited the expected N-glycosylation profile with one predominant glycoform, compared to the more heterogeneous profile of the CHO cell-made counterpart. While all three ZV1 glycovariants demonstrated similar neutralization potency, their ADE activity for DENV and ZIKV infection was remarkably different, with ZV1 produced in WT plants that completely forwent ADE. Further analysis indicated that the antibody-dependent cellular cytotoxicity (ADCC) against ZIKV-infected cells was preserved for all three ZV1 glycoforms, with fucose-free ZV1 produced in ∆XF plants having higher activity. Moreover, the in vivo efficacy against ZIKV infection, as determined in a mouse model, was demonstrated for the plant-produced ZV1 glycovariant that forwent ADE.

## 2. Material and Methods

### 2.1. Production of ZV1 in N. benthamiana Leaves

The gene sequences of the variable region of the heavy chain (HC) and light chain (LC) for ZV1 (Accession numbers: 5JHL_H and 5JHL_L, 2A10G6) [26] were synthesized and fused to the coding sequence of human IgG1 HC constant region and kappa LC constant region, respectively. The resulting HC and LC coding sequences were cloned into MagnICON-based plant expression vectors and transformed into *Agrobacterium tumefaciens*, as described previously [27]. WT and ∆XF *N. benthamiana* plants were grown and agroinfiltrated with *A. tumefaciens* strains harboring the ZV1 HC and LC 3′ vectors with a bacterial OD_600_ HC/LC ratio of 4:1 according to the protocols we described previously [28].

### 2.2. Temporal Expression and Purification of ZV1 from WT and Glycoengineered Plants

*N. benthamiana* leaves agroinfiltrated by ZV1 LC and HC expression vectors were harvested 5, 6, 7, and 8 days post agroinfiltration (DPI) to evaluate the temporal expression profile of the mAb by using an ELISA that detected only the assembled form of mAbs as we previously described [29]. Briefly, total leaf soluble protein was obtained by homogenizing leaves in extraction buffer (50 mM Tris-HCl pH 7.5, 150 mM NaCl) and clarified by centrifugation at 15,000× *g* for 30 min at 4 °C. Microtiter plates were coated with a goat anti-human gamma HC antibody (Southern Biotech, Birmingham, AL, USA) and incubated with the plant protein extract. After incubation and washing, an HRP-conjugated anti-human-kappa LC antibody (Southern Biotech, Birmingham, AL, USA) was used for detection. A plant-produced IgG isotype control mAb (E16) was used as a reference standard. For functional evaluation experiments, larger-scale ZV1 variants were extracted from leaves at 8 DPI as described above and purified with a Protein A-based method we developed previously [30].

### 2.3. Production and Purification of ZV1 from Chinese Hamster Ovary Cells

For CHO cell expression, mammalian cell expression vector pcDNA3.1 (Thermo Fisher Scientific, Waltham, MA, USA) was used to construct ZV1 LC and HC expression cassettes which were transfected into CHO-K1 cells with Lipofectamine (Thermo Fisher Scientific) as we previously described [31]. In contrast to conventional CHO cells, CHO-K1 provides the flexible option to start the initial culture as adherent cells and then scale up the production of mAbs in a resuspension culture. The media of ZV1 transfected cell culture was clarified by spinning at 15,000× *g* for 30 min, and ZV1^CHO^ was directly purified from clarified media by protein A affinity chromatography (MabSelect resin, Cytiva, Marlborough, MA, USA) according to the manufacturer’s protocol.

### 2.4. N-Linked Glycan Analysis

Mass spectrometry (MS) was used to determine the N-glycosylation profiles of ZV1 variants, as described previously [32]. Briefly, purified ZV1 variants were separated by SDS-PAGE, and the HC was extracted from the gel, trypsin digested, and then analyzed with a liquid-chromatography-electrospray ionization-mass spectrometry (LC-ESI-MS) system (Orbitrap Exploris 480, Thermo Fisher Scientific, Waltham, MA, USA). Glycopeptides that consist of the peptide and the attached N-glycans with various N-acetylhexosamine (HexNAc) units, hexose (mannose, galactose, glucose, etc.), deoxyhexose (fucose), and pentose (xylose) residues were detected as a set of peaks. Glycopeptide peaks were identified by searching with FreeStyle 1.8 (Thermo Scientific, Waltham, MA, USA), and deconvolution was performed using the Extract function. Glycans were annotated according to the nomenclature of the Consortium for Functional Glycomics (http://www.functionalglycomics.org (accessed on 3 March 2023).

### 2.5. ZIKV and DENV Neutralization

Plaque reduction neutralization test (PRNT) assays were used to measure the neutralizing potency of ZV1 glycovariants against ZIKV and DENV, as described in detail previously [9,10]. Briefly, ZV1 glycovariants were serially diluted in a serum-free medium (Opti-MEM serum-free medium, Life Technologies). ZIKV (PRVABC59, ATCC# VR-1843) and DENV-2 (ATCC# VR-1584) were diluted in Opti-MEM to a working concentration of 100 plaque-forming units (PFU) per well. ZIKV or DENV-2 were then mixed with diluted mAbs and incubated for 1 hr. Subsequently, the virus-mAb mixture was transferred to a plate with confluent Vero cells (ATCC# CCL-81). After removing the medium, cells were overlaid with fresh medium with 0.8% agarose (for ZIKV) or 1% methylcellulose (for DENV-2) and incubated for an additional 3 (ZIKV) or 4 (DENV) days at 37 °C. Following incubation, cells were fixed with 4% paraformaldehyde (PFA, MilliporeSigma, MA) and then stained with 0.2% crystal violet (Sigma, Saint Louis, MO, USA). Percent (%) neutralization was calculated as: [(number of plaque per well without mAb) − (number of plaque per well with diluted mAb)/(number of plaque per well without mAb) × 100]. The half maximal effective concentration (EC_50_) of each mAb was calculated using GraphPad Prism software (Version 9.3).

### 2.6. Antibody-Dependent Enhancement Assay

The enhancing activities for DENV-2 and ZIKV infection by the mAb glycovariants were determined with FcγRIIA^+^ K562 cells (ATCC # CCL-2243) as described previously [33]. ZV1 glycovariants were serially diluted, mixed with DENV-2 or ZIKV, and incubated for 1 h at 37 °C, and then mixed with K562 cells (MOI = 1). After 48 h (DENV-2) or 72 h (ZIKV) of incubation, cells were fixed with 4% PFA, permeabilized with 0.1% saponin (Sigma), and stained with mAb 4G2 (ATCC# HB112) conjugated to Alexa 488 (Invitrogen, Waltham, MA, USA). Flow cytometry was then performed with a Navios flow cytometer (Beckman Coulter) to determine the percentage of DENV or ZKIV-infected cells.

### 2.7. Antibody-Dependent Cellular Cytotoxicity (ADCC) Assay

Human peripheral blood mononuclear cells (PBMCs) were isolated from normal human buffy coat (BIOIVT.com), and NK cells were expanded through a 21-day period according to a published protocol [34]. NK cells were cultured in RPMI medium (Thermofisher) with 10% fetal bovine serum (FBS, Sigma) plus 50 units/mL human IL-2 (Sigma) for 24 h. NK cells (50,000 cells/well) were then plated in a 96-well V bottom TC plate (Thermofisher) at the density with E/T ratio 5:1. Vero cells were infected with ZIKV (MOI = 0.05) for 4 days, rinsed with PBS and then dislodged using a cell scrapper in PBS with 8 mM EDTA (Sigma). Uninfected Vero cells were used as a negative control and dislodged in parallel. Dislodged cells were resuspended in MEM media with 5% FBS at 1 million/mL and loaded with 5 µg/mL Calcein AM (Invitrogen) at 37 °C for 1 h. Infected or uninfected Vero cells were then resuspended in RPMI with 10% FBS at 0.1 million/mL and incubated with mAb variants (20 µg/mL) or a human IgG isotype control for 15 min before adding to the TC plate to mix with NK cells. The assay plate was centrifuged at 100× *g* for 1 min to initiate the assay. After 4-h incubation at 37 °C, the plate was centrifuged at 100× *g* for 5 min. The supernatant of the assay plate was then transferred to a new 96-well black/clear bottom plate (Thermofisher) for fluorescence reading (excitation 485 nm, emission 530 nm) in a SpectraMax M5 fluorometer (Molecular Device). Supernatant from wells that contain Vero cells only (no NK cells or mAb) and treated with 100 µL of 2% Triton X-100 (ThermoFisher) serves as the “maximum release” control. Supernatant from wells that contain Vero cells only and treated with 100 µL of RPMI media serves as the “spontaneous release” control. The % of mAb-NK cell-mediated cell lysis was calculated according to the formula [(test release-spontaneous release)/maximum release] × 100. Each sample was measured in triplicate.

### 2.8. Mouse Studies

Five-week-old type I interferon receptor (IFNAR)-deficient (*Ifnar1^−/−^*) mice with a C57BL/6J background (n = 10) were purchased from the Jackson Laboratory (Bar Harbor) and infected by subcutaneous inoculation on the ventral side of the right hind footpad with 1 × 10^5^ PFUs of ZIKV as previously described. In contrast to wild-type adult mice that are resistant to ZIKV infection, *Ifnar1^−/−^* mice are highly susceptible to ZIKV infection and develop disease due to their deficiency in IFNARs that abrogates their resistance to ZIKV infection via type I interferons. Mice were intraperitoneally treated with 470 µg of pZV1^WT^ mAb or PBS at 24 h post-ZIK−V infection. On day 3 post-infection, blood was collected in Trizol, and the level of viremia was quantitated by qPCR. Mice were observed daily for survival for 20 days post-infection.

### 2.9. Real Time-Quantitative PCR (RT-qPCR)

RT-qPCR was performed as described previously. In brief, TRI-reagent (Molecular Research Center Inc., Cincinnati, OH, USA) and iSCRIPT cDNA synthesis kit (Bio-Rad, Hercules, CA, USA) were used to isolate total RNA from mouse blood and to synthesize the first-strand complementary DNA (cDNA). ZIKV E protein and cellular *β*-actin RNA copy numbers were determined by probe-based (Bio-Rad) RT-qPCR in a CFX96 Real-Time system (Bio-Rad) using iTAQ^TM^ polymerase supermix with primers previously described. Viral copy numbers were expressed as PFU equivalent per milliliter of blood. PFU equivalents were determined by comparing the experimental RT-qPCR cycle threshold values (CT) to that of a standard curve generated by using RNA from Vero cells infected by 10-fold dilutions of ZIKV PRVABC59 stock virus of known titer (2 × 10^6^ PFU/mL), run as technical duplicates.

### 2.10. Statistical Analyses

GraphPad Prism software version 9.3 (GraphPad) was employed for data analysis. Specifically, one-way ANOVA and T-test were used to compare ADCC activities between different mAb variants. T-test was used to compare blood viral copy numbers between mouse groups treated with pZV1^WT^ and PBS. A *p*-value of <0.05 indicated a statistically significant difference.

## 3. Results

### 3.1. ZV1 Expression in Nicotiana benthamiana Plants

**Figure 1:**
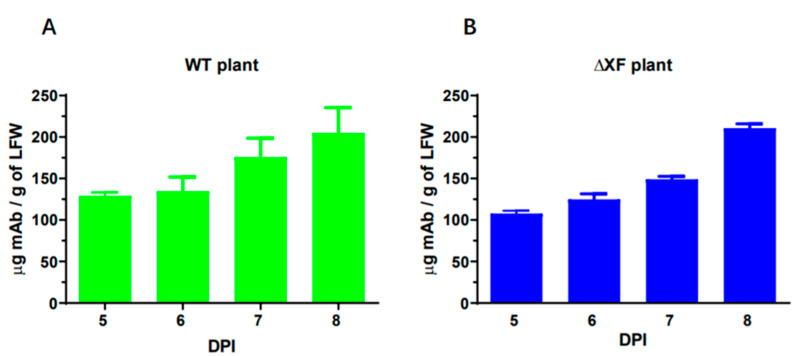
Temporal expression of ZV1 in *N. benthamiana* leaves. Leaves of WT (**A**) and ΔXFT (**B**) *N. benthamiana* plants were agroinfiltrated with ZV1 light chain and heavy chain expression vectors, and total leaf proteins were extracted on 5–8 DPI. MAb accumulation levels were quantitated by an ELISA that detects only assembled forms of IgG. Mean ± standard deviation (SD) from three independent infiltrations with technical triplicates are presented.

ZV1 was produced in *N. benthamiana* leaves via transient expression, and the expression levels were quantified by an ELISA that only detects the fully assembled form of ZV1. As shown in Figure 1, ZV1 was produced quickly and reached the highest level of accumulation on 8 DPI in both WT (Figure 1A) and ∆XF (Figure 1B) *N. benthamiana* plants, with an average level of ~205 and ~211 μg/g leaf fresh weight (LFW), respectively. This level of expression is similar to that previously reported for non-codon-optimized mAbs produced in plants [35,36]. Further characterization indicated that the plant-made ZV1 variants shared the equivalent sizes of HC and LC and assembled properly into the tetrameric IgG as CHO-cell-produced ZV1 (Figure S1).

### 3.2. N-Linked Glycosylation Pattern of ZV1 Produced in Plants and CHO Cells

We next examined the N-glycosylation of ZV1 produced in CHO cells and in different plant lines by LC-ESI-MS as the nature of the glycans has been shown to affect IgG’s Fc domain-mediated functions [37]. ZV1 produced in WT plants (ZV1^WT^) exhibited the expected complex-type N-glycans that terminate with N-acetylglucosamine residues with xylose and 1,3-linked core fucose (GnGnXF_3_, Table 1). ZV1 produced in ∆XF plants (ZV1^∆XF^) carried the mammalian type GnGn structures lacking xylose and fucose (Table 1). Notably, both ZV1^WT^ and ZV1^∆XF^ showed a single predominant N-glycoform with high degrees of homogeneity (79 and 88%, respectively) with a minor collection of high mannose glycoforms. ZV1 produced in CHO cells (ZV1^CHO^) displayed two major peaks of core α1,6 fucosylated structures with and without terminal β1,4-galactose (GnGnF_6_ and AGnF_6_) (Table 1).

Heavy chains of ZV1^CHO^, ZV1^WT^, and ZV1^∆XF^ were extracted after SDS-PAGE, trypsin digested, and analyzed by mass spectrometry (LC-ESI-MS). Glycopeptide peaks were identified using FreeStyle 1.8, and percentages of glycoforms were assigned based on approximate molar ratios from the peak heights. Consortium for Functional Glycomics nomenclature was used for annotation.

### 3.3. Neutralization of ZIKV and DENV by ZV1 Glycovariants

**Figure 2:**
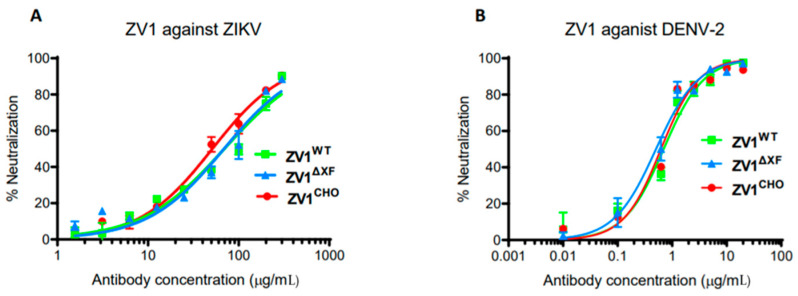
Neutralization of ZIKV and DENV by ZV1 mAb glycovariants. Serial dilutions of ZV1 mAb glycovariants were co-incubated with 100 PFU of ZIKV (**A**) or DENV-2 (**B**) and Vero cells for 3 (ZIKV) or 4 (DENV-2) days. Plaques were counted, and percent neutralization and EC_50_ were calculated. Experiments were performed at least twice with technical triplicates for each sample. Bars represent the SD of the mean.

A PRNT assay was performed to evaluate the potential of ZV1 glycovariants in neutralizing ZIKV and DENV. Our results showed that both plant-produced (ZV1^WT^ and ZV1^∆XF^) and CHO cell-produced (ZV1^CHO^) glycovariants exhibited strong neutralizing activity against both ZIKV and DENV with similar potency between the three glycovariants (Figure 2). Specifically, the concentration of 50% maximal neutralization effect (EC_50_) value of ZV1^WT^, ZV1^∆XF^, and ZV1^CHO^ was 69.34, 68.86, and 51.19 µg/mL for ZIKV (Figure 2A), and 0.69, 0.51, and 0.62 µg/mL for DENV (Figure 2B), respectively.

### 3.4. Antibody-Dependent Enhancement of DENV and ZIKV Infection by ZV1 Glycovariants

**Figure 3:**
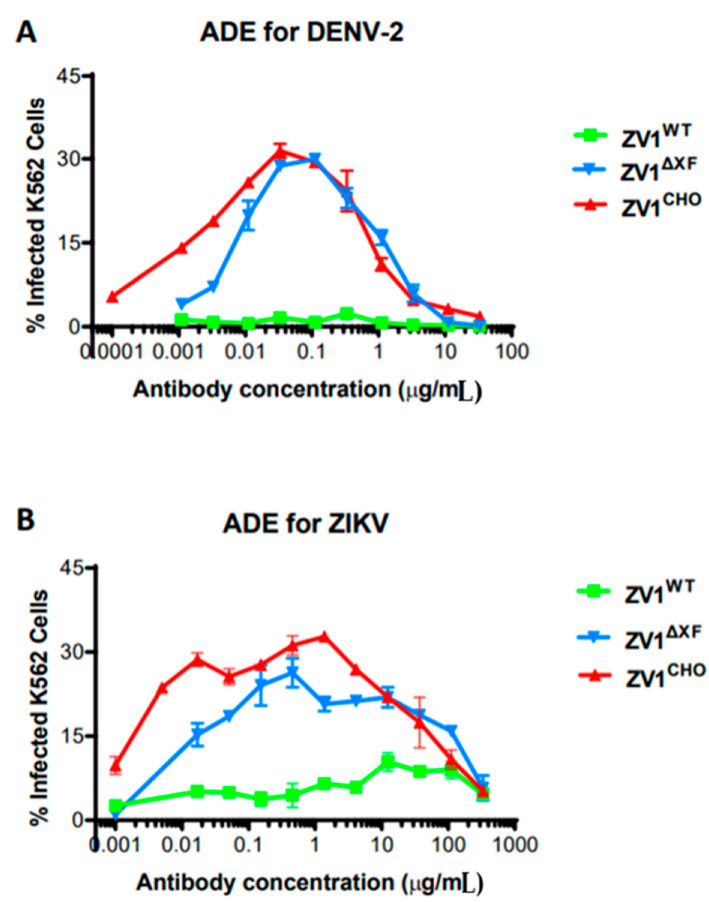
Antibody-dependent enhancement (ADE) of ZV1 glycovariants for DENV and ZIKV infection. Serial dilutions of ZV1 glycovariants were mixed with DENV-2 (**A**) or ZIKV (**B**) and then incubated with FcγRIIa^+^ K562 cells. After incubation, cells were fixed, permeabilized, and stained with an anti-flavivirus E antibody and then analyzed by flow cytometry to identify DENV-2 or ZIKV-infected cells. Results (Mean ± SD) from at least two independent experiments with technical triplicates for each sample are presented.

The potential impact of N-linked glycosylation variation on the enhancement of DENV and ZIKV infection by ZV1 was investigated in an in vitro assay. As shown in Figure 3, ZV1^CHO^ triggered ADE of DENV (Figure 3A) infection in K562 cells that express the human FcγRIIa, a phenomenon observed for CHO-cell-produced mAbs in previous studies [31,38]. Similarly, ZV1^CHO^ also promoted the ADE of ZIKV effectively (Figure 3B). ZV1^∆XF^ also exhibited ADE activity for DENV and ZIKV comparable to that of ZV1^CHO^ (Figure 3). In contrast, ZV1^WT^ displayed no ADE activity for DENV (Figure 3A, *p* < 0.05 compared to the curve of ZV1^CHO^) and greatly reduced ADE for ZIKV infection (Figure 3B, *p* < 0.05 compared to the curve of ZV1^CHO^).

### 3.5. Antibody-Dependent Cellular Cytotoxicity of ZV1 Glycovariants

**Figure 4:**
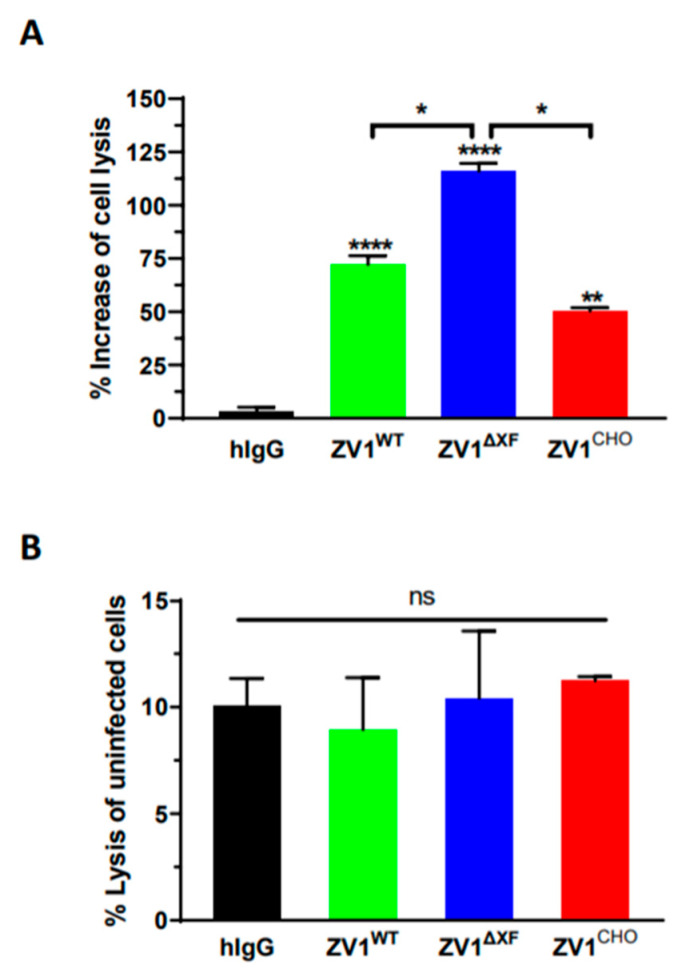
Antibody-dependent cellular cytotoxicity (ADCC) of ZV1 glycovariants. ADCC assay was performed by incubation of NK cells with ZIKV-infected Vero cells (**A**) or uninfected Vero cells (**B**) at the E:T ratio of 5:1 in the presence of indicated mAb glycovariants or a human IgG isotype negative control (hIgG). The % of mAb-NK cell-mediated Vero cell lysis (ADCC target cell killing) and % increase of ZV1 ADCC compared to the negative control hIgG or between ZV1 glycovariants were calculated. ****, **, * and ns indicate *p* values < 0.0001, =0.0021, <0.05, and >0.05, respectively.

We also investigated the ADCC of ZV1 variants aiming to identify an N-glycoform that forgoes ADE but still retains the Fc effector function. An ADCC assay was carried out to examine the effect of ZV1 Fc glycosylation on the killing of ZIKV-infected Vero cells by natural killer (NK) cells. Compared to the IgG isotype negative control (hIgG), all three glycovariants exhibited significantly enhanced NK-mediated ADCC activity against ZIKV-infected Vero cells (Figure 4A, ZV1^WT^ *p* < 0.0001; ZV1^∆XF^ *p* < 0.0001; ZV1^CHO^ *p* = 0.0021). Notably, ZV1^WT^ and ZV1^∆XF^ showed higher ADCC potency than that of ZV1^CHO^, with ZV1^∆XF^ having the highest activity with statistical significance (Figure 4A, ZV1^∆XF^ vs. ZV1^CHO^: *p* = 0.023; ZV1^∆XF^ vs. ZV1^WT^: *p* = 0.034). In comparison, when NK cells were incubated with non-infected Vero cells, no significant difference (*p* > 0.13) of target cell lysis was observed between the treatment using IgG isotype negative control or any of the three glycovariants (Figure 4B), confirming the lysis of ZIKV-infected cells is ADCC-specific.

### 3.6. Therapeutic Efficacy of Plant-Derived ZV1 against ZIKV Infection in Mice

**Figure 5:**
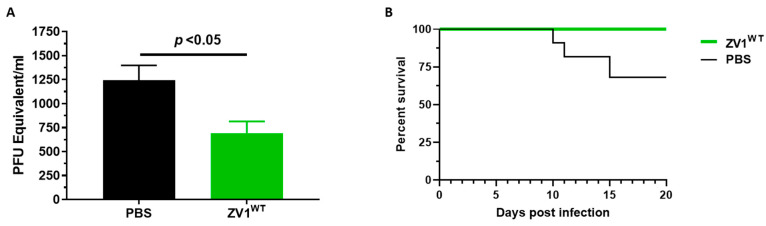
Therapeutic efficacy of plant-produced ZV1 glycovariant in mice. Five-week-old *Ifnar1^−/−^* mice were infected with 1 × 10^5^ PFU of ZIKV via footpad inoculation. After 24 h, mice were intraperitoneally injected with ZV1^WT^ or PBS (negative control). Mice were monitored daily for survival for 20 days post-ZIKV infection, and on day 3 post-infection, blood was collected for viral quantification. (**A**) The ZIKV genome copy numbers were quantified by measuring ZIKV with qPCR and expressed as ZIKV PFU equivalent/mL of blood. (**B**) The survival curves of ZIKV-infected mice administered with ZV1^WT^ or PBS. The data were analyzed by a two-tailed Student’s *t*-test. The results are representative of two independent experiments and expressed as mean ± SEM.

Since ZV1^WT^ appears to forgo ADE of DENV infection but still preserves ADCC activity, we assessed its in vivo efficacy in a mouse model. Five-week-old type I interferon receptor-deficient (*Ifnar1^−/−^*) mice were inoculated in the footpad with 1 × 10^5^ PFU of ZIKV and administered intraperitoneally with 470 μg of ZV1^WT^ or PBS 24 h post-infection. Viremia at 3 days post-infection was measured by RT-qPCR. As shown in Figure 5A, mice treated with ZV1^WT^ developed significantly lower viremia compared to PBS-treated mice (*p* = 0.011). The survival of infected mice was further monitored daily for 20 days post-infection. The survival analysis showed that 100% of ZV1^WT^-treated mice survived compared to 70% of PBS-administered mice (Figure 5B). These results demonstrated the in vivo therapeutic efficacy of a mAb glycoform that forgoes ADE activity.

## 4. Discussion

The continued spread of the ZIKV epidemic and its association with severe abnormalities of human fetal development and neurological complications in adults call for the development of efficacious treatments. In the absence of licensed vaccines and antivirals, neutralizing mAbs, especially those against the ZIKV E protein, have become the prime therapeutic candidates to fight epidemics. For example, anti-ZIKV E mAbs have been shown to potently neutralize ZIKV, reduce tissue pathology, provide protection against lethal ZIKV challenge in vivo, decrease vertical transmission, and prevent ZIKV-induced microcephaly in mouse models [39,40,41]. While these mAbs hold great therapeutic promise, the risk of causing ADE and exacerbating diseases during subsequent infections from DENV or new strains of ZIKV impedes their clinical approval and application.

The development of ADE requires the specific interaction of the IgG Fc domain with FcγRs on the surface of host cells [17,42,43]. Therefore, interrupting the binding of antibodies to FcγRs is a major strategy to eliminate ADE. The most popular approach of this strategy is through Fc amino acid mutations to fully abrogate Fc-FcγR interaction [40,44]. However, this also eliminates Fc effector functions such as ADCC activity which has been shown to be required for the full potency of anti-DENV and certain anti-ZIKV antibodies [45,46]. This dilemma sparked our interest in fine-tuning the Fc-FcγR interaction so that ADE can be abolished while preserving other beneficial Fc effector functions. Since Fc-FcγR binding affinity depends on the composition of antibody Fc glycosylation [24], controlling antibody glycosylation is a potential way to fine-tune this specific interaction. However, it has been difficult to obtain mAbs that carry a homogenous N-glycan population for performing this type of research, as CHO cells usually produce glycoproteins with great glycan heterogeneity [47]. In contrast, plant glycoproteins generally exhibit one dominant N-glycoform [24]. Using this unique property of plants, glycoengineering has established multiple transgenic *N. benthamiana* lines that produce mAbs with various defined and homogenous mammalian N-linked glycans, thereby providing a favorable system for examining the effect of carbohydrate moieties on Fc-mediated functions [48].

Taking advantage of these plant lines, we produced two N-glycovariants of ZV1, a mAb against a pan-flavivirus epitope on the EDII fusion loop in plants along with the parent glycoform in CHO cells to investigate the effects of antibody glycosylation on its ADE risk. ZV1 was produced in two plant lines quickly within 8 days of gene introduction, and the two plant-derived ZV1^WT^ and ZV1^∆XF^ exhibited the expected yet distinct glycosylation profile with a high degree of homogeneity compared to the more heterogeneous profile of ZV1^CHO^. As expected, the difference in N-glycosylation among the three ZV1 variants did not alter their neutralization activity as both ZV1^WT^ and ZV1^∆XF^ demonstrated potent neutralizing potency as ZV1^CHO^ against both ZIKV and DENV. Interestingly, the three ZV1 glycovariants showed drastically different activity in trigging ADE of DENV and ZIKV infection in FcγR-expressing K562 cells. CHO-cell-produced ZV1^CHO^ promoted strong ADE activity, as observed previously for mammalian cell-derived anti-flavivirus mAbs [31,49]. ZV1^∆XF^ also induced ADE similarly to ZV1^CHO^. Notably, pZV1^WT^ appeared to abolish its ADE activity in contrast to the other two glycovariants. Specifically, the overall ADE activity of ZV1^WT^ was greatly reduced for both DENV and ZIKV compared to that of ZV1^CHO^ with statistical significance. However, the degree of ADE reduction by ZV1^WT^ was more noticeable for DENV than for ZIKV. For example, at the respective EC_50_ concentrations, ADE reduction by ZV1^WT^ appeared to be much more drastic for DENV than ZIKV. This may reflect the intrinsic difference in ADE activity between the two viruses [50]. One possible explanation is that DENV is more prone to enhance viral replication once being up-taken into host cells via ADE [18] and, therefore, still exhibited substantial ADE at the EC_50_ concentration. In contrast, ZIKV may have lower replication potential under such conditions and exhibited low ADE activity at EC_50_, thereby giving a seemingly lower degree of ADE reduction by ZV1^WT^. The observed low ADE activities of ZIKV under high antibody concentrations are consistent with the lack of conclusive evidence of ADE for this virus in vivo [51]. Since the clinical relevance of ADE has been confirmed only for DENV infection, our demonstration of ADE abrogation for DENV may have a profound impact for the development of mAb-based therapy against flavivirus. As the three ZV1 glycovariants share the same polypeptide backbone, the difference in their ability to induce ADE is mostly likely due to the unique N-glycans on their Fc domain. These results suggest that at least for ZV1, N-glycoforms of AAF_6_, AGnF_6_, and GnGn carry the risk of ADE, while the GnGnXF_3_ glycan forgoes ADE. Although GnGnXF_3_ glycans have shown altered ADE for antibodies against other flavivirus and alphavirus [29,31], great caution should be taken before this conclusion can be generalized to all anti-ZIKV antibodies as many more mAb glycoforms need to be analyzed and their effects confirmed by in vivo studies.

One of the drawbacks of using Fc-silenced mutations to eliminate ADE is the full abrogation of Fc-FcγR interaction and hence the total abolishment of the Fc effector function. We investigated if modulation of N-glycans also silences Fc effector functions. Our results demonstrated that all three glycovariants exhibited strong NK-cell mediated ADCC activity against ZIKV-infected Vero cells. It is not surprising that ZV1^∆XF^ displayed enhanced ADCC activity as it has been shown that afucosylated antibodies have increased ADCC potency compared to the fucosylated orthologues [52]. However, the activity of ZV1^WT^ is particularly interesting as this mAb glycoform forgoes ADE while still preserving at least equivalent ADCC activity as CHO-cell-produced ZV1^CHO^. Further investigation using a relevant *Ifnar1^−/−^* mouse model indicated that pZV1^WT^ significantly reduced viremia and increased mouse survival, demonstrating its potential as a post-exposure ZIKV therapeutic.

How a particular glycoform such as GnGnXF_3_ lacks the risk of ADE yet still preserves ADCC activity is a complex issue. The exhibition of ADCC activity but lack of ADE suggests that ZV1^WT^ retains some affinity to FcγRIIIA but has reduced binding to ADE-causing FcγRs. However, the induction of ADE is much more complicated than just requiring Fc-mediated binding of the virus-antibody complex to FcγR alone, as downstream signaling post FcγR-binding has been shown to be critical for ADE induction [53,54,55]. For example, FcγRIIA or FcγRIIB share a similar Fc-binding ectodomain but have very different intracellular signaling domains. As a result, the binding of the DENV-immune complex to FcγRIIA induces ADE, but the same binding to FcγRIIB inhibits this activity [54,56]. Therefore, it is possible that N-glycan variation may impact both the binding of the immune complex to FcγR-expressing cells as well as the downstream signaling that is required for viral entry and replication. It is notable that ZV1^WT^ contains plant-enriched β1,2-xylose (X) and α1,3-fucose (F_3_). There used to be concerns that such a mAb glycoform might evoke undesirable immune responses in humans with adverse consequences. However, all human studies to date indicate that these N-glycans do not influence the overall immunogenic profile or efficacy of plant-produced protein therapeutics [57,58]. For example, results from Phase I and Phase II clinical trials with *N. benthamiana*-produced influenza vaccine showed that no subject developed an IgE response to xylose- or fucose-containing motifs, and no allergic/hypersensitivity response was detected in any subject, including subjects with pre-existing plant allergies [59,60]. In a more detailed analysis, a validated assay was first developed to specifically detect anti-plant glycan antibodies in human serum and then used to investigate the presence of these antibodies in a healthy human population and in Gaucher disease (GD) patients treated with taliglucerase alfa, a plant-made human glucocerebrosidase [58]. The results demonstrate that the incidence of anti-plant glycan antibodies in the general population is low and does not increase in GD patients after up to 30 months of treatment [58]. Further evaluation showed that the safety or efficacy of taliglucerase alfa was not affected by anti-plant glycan antibodies in GD patients [58]. We expand these results by demonstrating that controlling N-glycosylation is a feasible approach to modulate the ADE activity of mAbs while preserving beneficial effector functions.

Collectively, we report the modulation of ADE activity by controlling mAb Fc glycosylation and the successful identification of an antibody variant that forgoes ADE while maintaining effector function. Since ZV1 has been shown to neutralize all four serotypes of DENV, WNV, and YFV, in addition to ZIKV [26], our findings will have a broad impact beyond the ZIKV model. For example, an ADE-free ZV1 variant can be developed as a safe pan-flavivirus prophylactic and therapeutic to prevent and treat multiple flavivirus infections without the risk of predisposing patients to develop life-threatening symptoms in subsequent infection by a related flavivirus. The same approach may be applicable to develop safer mAb-based therapeutics for other ADE-prone viruses, including SARS-CoV-2. Furthermore, this strategy can be used to abrogate complement-mediated ADE that has been observed during Ebola [61] and human immunodeficiency virus (HIV) [62] infections. Overall, we demonstrate that mAb glycovariants with various homogenous N-glycosylation profiles can be quickly produced in plants to investigate the impact of carbohydrate moieties on the Fc-mediated functions, including pathogenic ADE or beneficial effector functions such as ADCC. This may spur new interest in developing novel mAb variants by glycoengineering beyond the current paradigm of Fc mutations.

## Figures and Tables

**Table 1:** N-glycosylation characterization of ZV1 variants.

Major N-glycan Species (in %)	Schematic Presentation	ZV1^CHO^ (%)	AV1^WT^ (%)	ZV1^∆XF^ (%)
**GnGn**	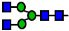			88
**GnGnX/GnGnXF_3_**	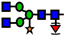		79	
**GnGnf_6_**	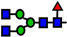	39		
**AGnF_6_**	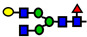	55		
**AAF_6_**	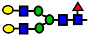	6		
**Man5-9**	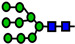		21	12

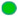
 Mannose, 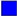 N-acetylglucosamine, 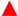 Fucose, 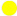 Galactose.

## Data Availability

The data presented in this study are contained within this article.

## Supplementary Materials

The following supporting information can be downloaded at: https://www.mdpi.com/article/10.3390/vaccines11040755/s1, Figure S1: Biochemical characterization of ZV1 glycovariants.
