# Supplementary material for: Plant-Produced Anti-Zika Virus Monoclonal Antibody Glycovariant Exhibits Abrogated Antibody-Dependent Enhancement of Infection
Source: Vaccines (Basel). 2023 Mar 29;11(4):755. doi: 10.3390/vaccines11040755 (PMC10144123; doi:10.3390/vaccines11040755)
Supplement: Supplementary file 1 [file vaccines-11-00755-s001.zip › vaccines-2289538-supplementary.pdf]

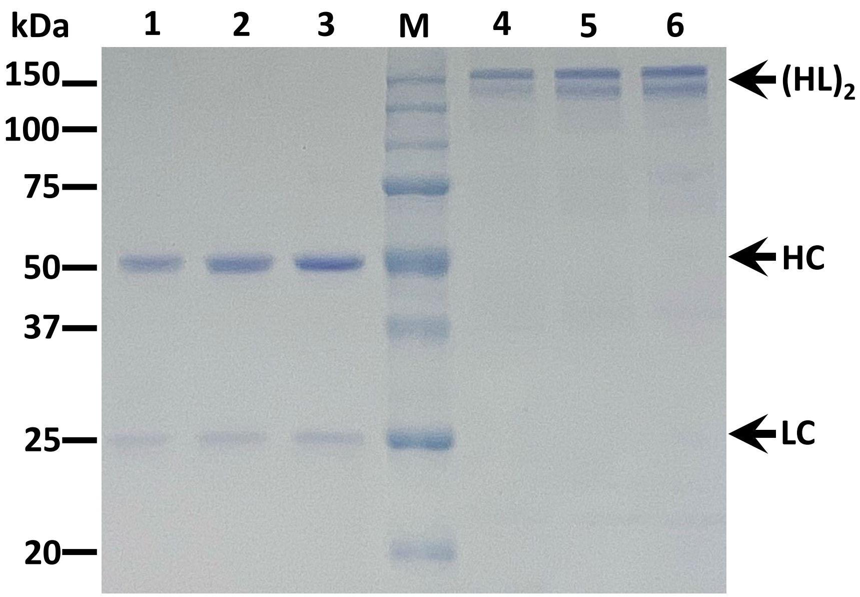

Figure S1: Biochemical characterization of ZV1 glycovariants.
ZV1 produced in WT (Lanes 1 and 4) and ΔXFT (Lanes 2 and 5) *N. benthamiana* plants and in CHO cells (Lanes 3 and 6) was purified by Protein A affinity chromatography. Purified ZV1 was subjected to SDS-PAGE under reducing (Lanes 1 - 3) or non-reducing conditions (Lanes 4 - 6) and total protein content was stained with Coomassie blue. LC: light chain. HC: heavy chain. $(HL)_2$: assembled heterotetrameric form of IgG. M: molecular weight marker One representative result of multiple experiments is shown.
